# Quantification of regional variation in ultra-processed food consumption and its sociodemographic correlates across Bangladesh, India, Pakistan, and Sri Lanka: insights from the South Asia Biobank

**DOI:** 10.1016/j.lansea.2025.100633

**Published:** 2025-07-18

**Authors:** Divya Bhagtani, Jean Adams, Fumiaki Imamura, Anwesha Lahiri, Rajendra Pradeepa, Sara Mahmood, Abu Ahmed Shamim, Fahmida Akter, Menka Loomba, Lathika Athauda, Laksara De Silva, Khadija I. Khawaja, Sajjad Ahmad, Vinitaa Jha, Anuradhani Kasturiratne, Prasad Katulanda, Malay K. Mridha, Ranjit M. Anjana, John C. Chambers, Nita G. Forouhi

**Affiliations:** a MRC Epidemiology Unit, Institute of Metabolic Science, University of Cambridge, School of Clinical Medicine, Cambridge, UK; b Madras Diabetes Research Foundation, Chennai, India; c Department of Endocrinology & Metabolism, Services Institute of Medical Sciences, Services Hospital, Lahore, Pakistan; d Centre for Non-Communicable Diseases and Nutrition (CNCDN), BRAC James P Grant of Public Health, BRAC University, Dhaka, Bangladesh; e Office of Research, Max Super Speciality Hospital (Devki Devi Foundation), New Delhi, India; f Department of Public Health, Faculty of Medicine, University of Kelaniya, Ragama, Sri Lanka; g Department of Clinical Medicine, Faculty of Medicine, University of Colombo, Colombo, Sri Lanka; h Punjab Institute of Cardiology, Lahore, Pakistan; i Department of Epidemiology and Biostatistics, School of Public Health, Imperial College London, London, UK; j Lee Kong Chian School of Medicine, Nanyang Technological University, Singapore

**Keywords:** Ultra-processed foods, Sociodemographic factors, South Asia

## Abstract

**Background:**

Sales of ultra-processed foods (UPFs) are rising in South Asia, yet UPF consumption and its sociodemographic determinants remain largely unknown. We aimed to quantify UPF consumption and investigate its sociodemographic correlates in four countries of South Asia.

**Methods:**

Between January 2020 and September 2022, the South Asia Biobank recruited 63,914 participants aged 18 years or older who were resident in Bangladesh, Pakistan, Sri Lanka, and North and South India, and self-reported as being of South Asian ethnicity. We analysed data from 60,714 eligible adults. Dietary consumption was assessed using interviewer-led 24-h recalls. Foods were classified by their degree of processing using the NOVA classification. Two-part multivariable-adjusted regression models examined associations of sociodemographic factors with the likelihood and quantity of UPF consumption.

**Findings:**

In Bangladesh, Sri Lanka, and North India, ∼75% of participants reported consuming UPFs during the previous day, versus 41% in South India and Pakistan. Among consumers, UPFs contributed 13–17% of total energy intake, with biscuits being a common source across regions. Other UPFs included sweetened beverages in Pakistan, packaged salty snacks in South India, and breakfast cereals in Bangladesh. Younger age was associated with UPF consumption in Pakistan and Sri Lanka whereas in Bangladesh and North India, older age was. Women were more likely to consume UPFs in all regions except Bangladesh. In Bangladesh, Pakistan, and North India, any level of education above none (i.e., primary, secondary, or higher) was associated with UPF consumption. Among consumers, UPF consumption was lower in married or cohabiting than single people, in all regions. UPF consumption was higher in rural versus urban residents in Bangladesh and Sri Lanka but lower in Pakistan.

**Interpretation:**

UPF consumption varied across South Asia by sociodemographic factors including age, gender, and education. Understanding this heterogeneity is crucial when designing interventions aimed at reducing UPF consumption. Our findings of regional variations in the types of UPFs consumed provide valuable insights for targeted interventions.

**Funding:**

The South Asia Biobank is funded by the 10.13039/501100000272National Institute for Health Research.

**Research in context**

**Evidence before this study**

Ultra-processed food (UPF) consumption is gaining global attention because of concerns about its potentially adverse health impacts, with growing interest in regions undergoing rapid socioeconomic changes, such as South Asia. A previously published systematic review conducted in 2023 on the relationship between sociodemographic characteristics and UPF consumption, using nationally representative cohorts from 32 countries, revealed a significant evidence gap with no studies conducted in South Asian countries. We conducted a systematic PubMed search up to March 9, 2024, using the terms: ‘ultra-processed’ OR ‘ultraprocessed’ OR ‘ultra-processing’ OR ‘ultraprocessing’ AND ‘South Asia’ OR ‘Bangladesh’ OR ‘Bhutan’ OR ‘India’ OR ‘Pakistan’ OR ‘Nepal’ OR ‘Sri Lanka’ OR ‘Afghanistan’ OR ‘Maldives’ and found no studies that reported on the sociodemographic factors influencing UPF consumption in South Asia. However, an updated search on Nov 27, 2024, identified two studies, one each from Bangladesh and India. The study from Bangladesh had a number of limitations: it lacked representativeness in terms of age and area of residence by focussing on solely rural adolescents (n = 2463); it did not assess associations with total UPF consumption, focussing instead on four specific types of UPF consumption; and the sociodemographic variables were limited to gender, household wealth, and maternal and adolescent education. The study from India was restricted to only urban young adults (n = 630) recruited from colleges, and it restricted analysis to high UPF consumption, defined as >13% of energy intake from UPFs without any justification for this definition. These limitations underscore the need for more comprehensive studies in South Asia.

**Added value of this study**

To our knowledge, this is the first study to assess the sociodemographic correlates of UPF consumption in South Asia, using individual-level dietary recall data in a large population-based study of South Asian adults. In five regions of South Asia, we quantified UPF consumption and identified commonly consumed UPFs in each region such as sweetened beverages in Pakistan, salty snacks in South India, breakfast cereals in Bangladesh, and biscuits across all regions. We observed that sociodemographic correlates of both UPF consumption and consumption level amongst consumers varied by region. In Pakistan and Sri Lanka, younger age was associated with UPF consumption whereas in Bangladesh and North India, older age was. Across all regions except Bangladesh, women were more likely to consume UPFs. Among UPF consumers, across all regions, married or cohabiting individuals consumed less UPFs than single individuals and in Bangladesh and Sri Lanka UPF consumption was higher in rural residents as compared to urban. Across all regions, being in paid employment was not associated with UPF consumption or consumption level amongst consumers.

**Implications of all the available evidence**

Prior evidence suggested that UPF consumption patterns vary by several sociodemographic factors across countries, but data were lacking for South Asia. Our findings suggest that sociodemographic factors influencing UPF consumption vary across regions of South Asia, emphasising that public health guidance should be tailored to regional contexts. In our study, factors such as being a woman and having any level of education compared to no education were associated with greater UPF consumption in some regions, suggesting that public health messaging and targeted interventions may need to vary by population subgroups. Further research is needed to confirm our findings. Similar studies in younger population groups, including, infants, children, and adolescents, will also be important to provide valuable public health insights.

## Introduction

Home to nearly a quarter of the global population, South Asia has a disproportionately high burden of non-communicable diseases (NCDs) such as type 2 diabetes and cardiovascular disease.^1^ The prevalence of diabetes in South Asia is projected to escalate over 150% from 2000 to 2035,^2^ reaching an estimated 121 million people with diabetes, twice as many as Europe.^2^^,^^3^ The rising prevalence of NCDs in South Asia can be attributed to a multitude of interlinked factors including but not limited to rapid urbanisation, globalisation, population ageing, increased physical inactivity, and proliferation of suboptimal diets.^4^

Suboptimal diets have been estimated to be responsible for one-fifth of the global mortality and continue to be a key contributor to the growing NCD burden.^5^ Diets high in ultra-processed foods (UPFs) have been recognised to be nutritionally inadequate.^6^ UPFs are industrial formulations made almost entirely from ingredients that have undergone a series of physical and often chemical processes that alter their properties to make them convenient, microbiologically safe, and highly palatable.^6^^,^^7^ These foods typically contain additives with little or no intact or whole foods (e.g., crisps, biscuits, candies, and carbonated drinks).^7^ Several studies have characterised UPF-rich diets to be high in added sugar, sodium, saturated fat, and trans-fat and found them to be obesogenic.^8^^,^^9^ A growing body of evidence summarised in three systematic reviews and meta-analyses indicated that, compared to those reporting low UPF consumption, adults with higher UPF consumption may be at a greater risk of obesity, metabolic syndrome, cardiovascular disease, and death.^8^^,^^10^^,^^11^ Despite critical interest, most research on the role of UPF consumption in NCDs^10^^,^^11^ has focused on Western populations. A notable exception is the 21-country Prospective Urban Rural Epidemiology (PURE) study, which included data from three countries in South Asia (Bangladesh, India, and Pakistan).^12^^,^^13^ Of two reports on UPF consumption from the PURE study, one examined the association between UPF intake and risk of inflammatory bowel disease but found no significant association in South Asia,^12^ while the other assessed the association between UPF consumption and mortality without reporting region-specific findings,^13^ further highlighting the need for region-specific research.

Compared to high-income countries, growth in sales of UPFs has been higher in low- and middle-income countries.^14^ For example, India and Pakistan recorded the highest annual growth rate of UPF sales between 2009 and 2019, despite their low absolute sales levels.^15^ While the surge of UPFs in South Asia is acknowledged, research on the sociodemographic factors influencing UPF consumption remain limited, as summarised in a 2023 systematic review.^16^ Only two studies, one each from Bangladesh^17^ and India,^18^ have assessed the association between sociodemographic factors and UPF consumption, but both were limited in representativeness and scope, focussing on specific population subgroups and types of UPF consumption. Population-based studies are needed for South Asia to inform targeted public health interventions and tailoring of strategies to address the unique challenges faced by different groups of people. We aimed to quantify UPF consumption and investigate the relationship between sociodemographic characteristics and UPF consumption, using detailed dietary intake data from five regions of South Asia.

## Methods

### Study population and data collection

**Fig. 1:**
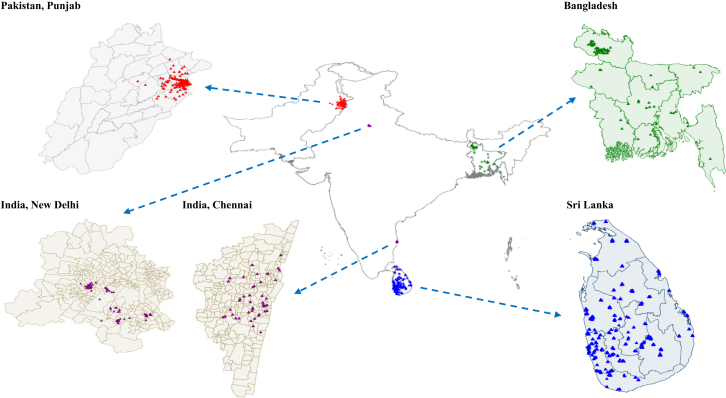
**Locations of the South Asia Biobank (SAB) surveillance sites**.

The South Asia Biobank (SAB) is an ongoing population-based cohort of South Asian adults living in five regions: Bangladesh, Pakistan, Sri Lanka, North India, and South India (Fig. 1). Details about sampling and recruitment have been published previously.^19^ Briefly, SAB was established to investigate the genetic and environmental determinants of NCD risks in South Asians. Between January 2020 and September 2022, the SAB recruited 63,914 participants aged 18 years or older who self-reported as being of South Asian ethnicity. Participants were recruited from 118 surveillance sites using existing infrastructure such as local primary community healthcare centres. The resident population was identified using national administrative data, existing household listings, house-to-house visits, and reviews of government census data. Within each household, all eligible individuals were invited to participate in the study. Exclusion criteria included pregnancy, non-permanent residence (<12 months) in the surveillance site, and serious illness expected to reduce life expectancy to less than 12 months. This study was conducted according to the guidelines in the Declaration of Helsinki. Research approval was obtained from the Imperial College London Ethics Committee (ref: 18IC4698) and the local institutional review boards in each of the participating countries. Written informed consent was obtained from all participants.

Participants attended the study surveillance sites for baseline assessments of sociodemographic, anthropometric, behavioural (including diet and physical activity), clinical, and biological measurements. In the current study, we evaluated 60,714 participants in total after excluding those who provided invalid dietary data (n = 3200), designating data to be invalid if estimated energy intake was <800 or >4200 kcal/day for men and <500 or >3500 kcal/day for women.

### Sociodemographic characteristics

Sociodemographic data were collected using a standardised questionnaire.^19^ The sociodemographic variables considered in this study were: age (18–39 years [y], 40–59 y, and ≥60 y), gender, area of residence (rural or urban), level of education, employment (in paid employment vs not), marital status (married or cohabiting vs never married, separated, divorced, or widowed), household income, and number of people in the household. Education was assessed based on the highest level of education completed and was categorised as no formal schooling or below primary, primary, secondary, and higher than secondary school education. Income was recorded as average monthly household income, in local currency. For comparability of income between South Asian countries, income was converted to US dollar, adjusted for purchasing power parity (PPP) and categorised into tertiles.

### Dietary assessment and classification of food by the extent and purpose of processing

Dietary data were collected using the digital 24-h recall tool (Intake24) adapted for dietary assessment in South Asian populations. Interviewers administered a single dietary recall with Intake24 to record all food and beverages consumed by the participant during the previous day. The quantity of each item consumed was also recorded in Intake24 which automatically generated the corresponding intake estimates of energy and nutrients. Further details about the Intake24 tool have been previously published.^20^ The estimates of total energy intake (kcal/day) were used to calculate relative energy (%kcal) from UPFs.

Two authors (DB and AL), with knowledge of South Asian foods, independently classified each of the 2283 food items within the South Asia version of Intake24 into one of four mutually exclusive groups based on their nature, extent, and purpose of processing, following the NOVA classification system^7^: group 1) unprocessed and minimally processed foods (e.g., fruits, vegetables, legumes, milk, eggs, meats); group 2) processed culinary ingredients (e.g., salt, sugar, honey, vegetable oils, butter); group 3) processed foods (e.g., canned and bottled fruits or vegetables or legumes in brine, salted or sugared nuts and seeds, smoked fish and meats); or group 4) ultra-processed foods (e.g., carbonated drinks, sweet or salty packaged snacks, candies, cookies). Supplementary Table S1 presents further details and examples of the NOVA classification framework. Single foods (e.g., apple, carrot, bread) were systematically assigned to their respective NOVA group. Mixed dishes were classified based on their main ingredient and were considered to be unprocessed and minimally processed foods because mixed dishes in South Asia are predominantly freshly made dishes prepared by combining unprocessed and minimally processed foods with culinary ingredients and without any additives. A similar approach has been used previously.^9^^,^^21^, ^22^, ^23^ Discrepancies regarding the degree of processing were resolved by discussion and erring on the side of classifying as not ultra-processed.

### Statistical analysis

Visualisation and preliminary analyses of the UPF-related dependent variable % UPF kcal per day, indicated that the assumptions of linear regression could not be satisfied because of skewed distribution and many zero values (Supplementary Figure S1). Therefore, we used two-part models^24^ to examine the relationship between sociodemographic factors and each of (i) the likelihood of consuming UPF (i.e., binary) in the total population and (ii) the level or quantity of UPF consumption (continuous) among consumers. For the first part (likelihood of UPF consumption) with a binary dependent variable of UPF consumption (yes/no), we fitted a logit regression model. For the second part (among consumers), since the distribution of the outcome variable remained skewed even after zero values were removed, we fitted a regression model with a gamma family and a log link function. The effect measures from the first part of the regression model are odds ratios (OR), while those from the second part are relative differences in the amount of UPF intake. Coefficients from both models were exponentiated to aid interpretation.

For the analysis of sociodemographic correlates of UPF consumption (overall and in consumers only), two-part multivariable models were built. Proportion of energy intake derived from UPFs was the dependent variable, and sociodemographic factors were the simultaneous explanatory variables. Models were built separately for each of the five SAB regions. Models were mutually adjusted for all sociodemographic factors including age groups, gender, area of residence, level of education, employment, marital status, income, number of people in the household, and study site. Additionally, behavioural factors such as smoking (including both smoke and smokeless tobacco products) and physical activity were also adjusted for in these models. Alcohol consumption was reported by 0.6% of participants and hence was not included as a covariate in the models due to near-zero variance. Total energy intake was adjusted for in the multivariable models to obtain results for interpretation of diet quality independent of diet quantity and with lesser correlated measurement errors.^24^ The same covariates were used in each part of the two-part models. To obtain overall SAB cohort estimates and explore the heterogeneity in observed associations by region, estimates from each region were pooled using an inverse-probability weighted meta-analysis model. The I^2^ statistic was used to express heterogeneity, where we considered I^2^ ≥ 75% as substantial heterogeneity between the regions.^25^ All analyses were conducted using the statistical software package Stata version 17.0 (Stata Corp Ltd., Texas, USA). A two-sided p < 0.05 was considered statistically significant.

### Role of the funding source

The funder of the study had no role in study design, data collection, data analysis, data interpretation, or writing of the report. The corresponding author had full access to all the data in the study and had final responsibility for the decision to submit for publication.

## Results

Among 60,714 participants the mean age was 46.4 (SD 13.9) years and 62.7% were women. Across SAB regions, approximately 75% of the participants in Bangladesh, Sri Lanka, and North India reported consuming UPFs during the previous 24-h. In Pakistan and South India, this figure was 41%.

Table 1 presents the proportion of participants consuming UPFs, stratified by sociodemographic and behavioural characteristics across five South Asian regions. UPF consumption was higher among older adults (aged ≥60 years) in Bangladesh and North India, while in Pakistan, Sri Lanka, and South India, younger adults (aged 18–39 years) represented a larger share of UPF consumers. In all regions except Sri Lanka, a higher proportion of UPF consumers were men than women. In Bangladesh and Pakistan, UPF consumption was more prevalent among urban residents than rural, whereas in Sri Lanka, a greater proportion of UPF consumers resided in rural areas. Across all regions, UPF consumption was more common among participants with higher educational attainment, particularly those with secondary and higher education, compared to those with none or below primary education. A greater proportion of UPF consumers were in paid employment across all regions. In Bangladesh, Pakistan, and North India, a greater proportion of UPF consumers were in the highest tertile of household income, while in Sri Lanka and South India, the lowest income tertile had the highest proportion of UPF consumers. Among UPF consumers, the proportion reporting insufficient physical activity (<150 min/week) ranged from 81.3% in Bangladesh to 39.9% in Pakistan. Smoking status and household size did not show a consistent pattern across regions.

**Table 1:** Proportion consuming ultra-processed foods (UPFs) by participant characteristics, stratified by country or region: results from the South Asia Biobank, n = 37,781 (UPF consumers only).

	Bangladesh	Pakistan	Sri Lanka	North India	South India
	(n = 11,384)	(n = 6894)	(n = 14,038)	(n = 2934)	(n = 2531)
**Sociodemographic characteristics**					
**Age**					
18–39 years	72.4	46.3	77.9	73.9	44.9
40–59 years	76.3	38.9	75.8	74.4	41.4
≥60 years	78.4	37.0	71.8	75.3	39.4
**Gender**					
Women	69.0	39.2	76.0	73.2	41.2
Men	82.1	44.6	74.0	76.2	41.6
**Area of residence**					
Rural	74.2	39.4	78.2	NA	NA
Urban	75.9	41.4	73.4	74.3	41.4
**Education level**					
None or below primary	72.0	35.3	73.6	68.4	40.5
Primary	75.5	39.1	74.6	73.9	41.2
Secondary	78.8	46.0	75.5	77.3	41.7
Higher than secondary	82.5	55.0	77.8	76.4	42.7
**Employment**					
Not in paid employment	70.0	39.3	75.3	73.6	41.1
In paid employment	80.5	45.5	75.4	75.3	41.6
**Marital status**					
Never married, separated, divorced, or widowed	72.5	44.1	76.5	75.4	40.9
Married or cohabiting	75.1	40.5	75.1	74.0	41.5
**Income tertiles in US$ PPP**					
First tertile—Lowest	72.3	39.0	76.3	73.5	47.0
Second tertile—Middle	75.3	38.9	75.6	71.4	42.9
Third tertile—Highest	80.2	48.5	74.6	76.0	35.3
**No. of people in the household**					
1	66.7	53.7	72.8	64.6	40.5
2	74.6	48.5	76.7	80.0	43.5
3	74.6	42.6	77.3	75.4	43.9
4	73.9	44.0	75.0	74.5	39.9
>4	76.0	40.3	74.2	73.1	40.0
**Behavioural characteristics**					
**Smoking**					
Never smoked	67.4	40.9	75.2	74.4	41.4
Current smoker	80.8	43.7	75.7	72.4	39.5
Ex-smoker	81.5	24.0	76.7	77.9	45.8
**Physical activity**					
% with insufficient physical activity (<150 min of moderate-intensity activity per week)	81.3	39.9	74.4	74.1	44.4

UPF—ultra-processed food, PPP—purchasing power parity, NA—not applicable due to unavailable data.

### Distribution of dietary consumption according to NOVA food groups

**Fig. 2:**
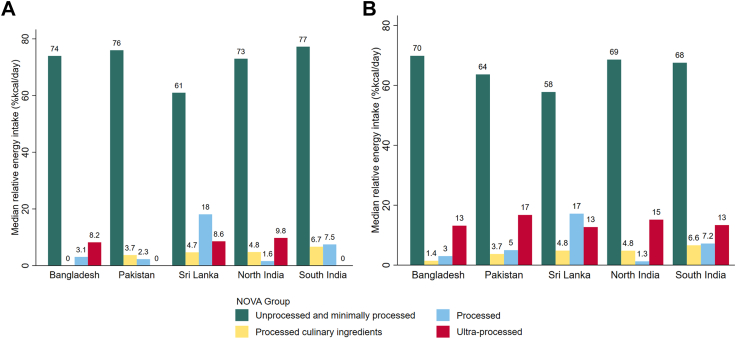
a: **Distributionof dietary consumption by NOVA food groups, in South Asia, among all participants (including consumers and non-consumers of ultra-processed foods)**. Results from the South Asia Biobank, n = 60,714 (Bangladesh, n = 15,212; Pakistan, n = 16,804; Sri Lanka, n = 18,634; North India, n = 3948; South India, n = 6116). b: **Distribution of dietary consumption by NOVA food groups, in South Asia, among consumers of ultra-processed foods**. Results from the South Asia Biobank, n = 37,781 (Bangladesh, n = 11,384; Pakistan, n = 6894; Sri Lanka, n = 14,038; North India, n = 2934; South India, n = 2531).

Mean daily energy intake varied across regions, with 1940 (SD 736) kcal/day in Bangladesh, 1891 (682) in North India, 1721 (661) in Sri Lanka, 1405 (488) in South India, and 1364 (544) in Pakistan. The assignment of NOVA groups between the two coders in the current research showed a strong inter-rater match, with over 86% agreement. Unprocessed and minimally processed foods made the greatest contribution to dietary intakes in all SAB regions (Fig. 2a). Across all participants (including consumers and non-consumers of UPFs), the median contribution of UPFs to dietary energy intake (%kcal) varied across regions, with North India at 10%, followed by Sri Lanka (9%), Bangladesh (8%), Pakistan (0%), and South India (0%). The reported 0% median UPF intake in Pakistan and South India reflects the distribution of intake in these populations, where a large proportion of participants (∼60%) reported no UPF consumption on the day of dietary recall (Supplementary Figure S1). Among UPF consumers only (Fig. 2b), the median contribution of UPFs to dietary energy intake (%kcal) was highest in Pakistan (17%) followed by North India (15%), Sri Lanka (13%), South India (13%), and Bangladesh (13%). Biscuits were the most common UPF source across all SAB regions among UPF consumers. In addition to biscuits, other UPFs among consumers included: sweetened beverages in Pakistan, packaged salty snacks in South India, and breakfast cereals in Bangladesh (Table 2).

**Table 2:** Distribution of dietary consumption according to NOVA food groups: results among consumers of ultra-processed foods from the South Asia Biobank, n = 37,781.

NOVA food groups expressed as kcal/day	Bangladesh (n = 11,384)	Pakistan (n = 6894)	Sri Lanka (n = 14,038)	North India (n = 2934)	South India (n = 2531)
	Median (IQR)	Median (IQR)	Median (IQR)	Median (IQR)	Median (IQR)
**Unprocessed and minimally processed foods**	1187 (810, 1612)	846 (604, 1142)	895 (588, 1261)	1193 (868, 1591)	940 (729, 1204)
**Processed culinary ingredients**	31 (0, 71)	62 (0, 123)	86 (31, 142)	90 (48, 145)	93 (62, 139)
**Processed foods**	52 (0, 440)	76 (0, 310)	282 (113, 581)	25 (0, 250)	107 (0, 242)
**Ultra-processed foods**	227 (110, 470)	222 (125, 385)	209 (110, 373)	257 (144, 496)	189 (100, 305)
Pastries, buns, & cakes; Confectionary; Industrial desserts (incl. ice-cream)	0 (0, 0)	0 (0, 0)	0 (0, 0)	0 (0, 0)	0 (0, 0)
Biscuits	0 (0, 56)	0 (0, 147)	54 (0, 145)	47 (0, 147)	0 (0, 147)
Industrialised packaged breads; Deep-fried local breads; Deep-fried local snacks	0 (0, 0)	0 (0, 0)	0 (0, 0)	0 (0, 0)	0 (0, 0)
Savoury & sweet spreads; Sauces, syrups, & dressings	0 (0, 0)	0 (0, 0)	0 (0, 0)	0 (0, 0)	0 (0, 0)
Sugar-sweetened & artificially sweetened beverages	0 (0, 0)	0 (0, 46)	0 (0, 0)	0 (0, 0)	0 (0, 0)
Other ultra-processed foods	0 (0, 3)	0 (0, 0)	0 (0, 0)	0 (0, 0)	0 (0, 0)
Local sweets	0 (0, 0)	0 (0, 0)	0 (0, 0)	0 (0, 0)	0 (0, 0)
Packaged salty snacks	0 (0, 0)	0 (0, 0)	0 (0, 0)	0 (0, 0)	0 (0, 50)
Milk-based drinks	0 (0, 0)	0 (0, 0)	0 (0, 0)	0 (0, 0)	0 (0, 0)
Packaged pre-prepared meals; Pizza; Deep fried chips or French fries; Sausage & reconstituted meat products	0 (0, 0)	0 (0, 0)	0 (0, 0)	0 (0, 0)	0 (0, 0)
Breakfast cereals	0 (0, 110)	0 (0, 0)	0 (0, 0)	0 (0, 0)	0 (0, 0)

Values are median and interquartile range (IQR).

### Sociodemographic correlates of the likelihood of ultra-processed food consumption

Variation in the associations was seen between sociodemographic factors and UPF consumption across SAB regions in the total sample including consumers and non-consumers (n = 60,714) (Table 3). Younger age (18–39 years) was significantly associated with UPF consumption in Pakistan and Sri Lanka whereas in Bangladesh and North India, older age was. In all regions except Bangladesh, women were more likely to report UPF consumption than men. In Sri Lanka, rural residence (vs urban) was associated with UPF consumption, but in other regions there was no significant association with area of residence. Any level of education (compared to none or below primary) and higher household income was significantly associated with UPF consumption in Bangladesh, Pakistan, and North India, but not in Sri Lanka and South India. Being in paid employment was not associated with UPF consumption in any SAB region (p > 0.05). Larger household size (≥2 people) was significantly associated with UPF consumption in Sri Lanka and North India, whereas in Pakistan single-person households were more likely to consume UPFs. Results from the meta-analysis indicated substantial heterogeneity (I^2^ ≥ 75%) in the associations between sociodemographic factors and UPF consumption across regions of South Asia (Supplementary Figure S2).

**Table 3:** Odds ratio for the association between sociodemographic characteristics and ultra-processed foods (UPFs) in South Asia, among all participants including consumers and non-consumers of UPFs: results from the South Asia Biobank, n = 60,714 (Bangladesh, n = 15,212; Pakistan, n = 16,804; Sri Lanka, n = 18,634; North India, n = 3948; South India, n = 6116).

UPFs expressed as %kcal/day	Bangladesh (74.8% UPF consumers)		Pakistan (41.0% UPF consumers)		Sri Lanka (75.3% UPF consumers)		North India (74.3% UPF consumers)		South India (41.4% UPF consumers)	
	OR [95% CI]	p value	OR [95% CI]	p value	OR [95% CI]	p value	OR [95% CI]	p value	OR [95% CI]	p value
Age: 40–59 years (ref: 18–39 years)	1.23 [1.12, 1.34]	<0.001	0.84 [0.78, 0.91]	<0.001	0.94 [0.86, 1.03]	0.006	1.15 [0.97, 1.36]	0.063	0.89 [0.76, 1.05]	0.177
Age: ≥60 years (ref: 18–39 years)	1.29 [1.12, 1.48]		0.86 [0.77, 0.95]		0.84 [0.76, 0.94]		1.32 [1.03, 1.68]		0.99 [0.82, 1.19]	
Gender: Men (ref: Women)	1.36 [1.18, 1.57]	<0.001	0.88 [0.80, 0.97]	0.011	0.72 [0.66, 0.79]	<0.001	0.87 [0.70, 1.07]	0.194	0.82 [0.72, 0.94]	0.005
Area of residence: Urban (ref: Rural)	1.06 [0.98, 1.16]	0.150	1.06 [0.95, 1.19]	0.302	0.87 [0.81, 0.94]	<0.001	NA		NA	
Education level: Primary (ref: None or below primary)	1.43 [1.31, 1.57]	<0.001	1.28 [1.16, 1.41]	<0.001	0.99 [0.88, 1.11]	0.610	1.24 [0.99, 1.55]	0.010	1.05 [0.90, 1.22]	0.847
Education level: Secondary (ref: None or below primary)	1.66 [1.41, 1.95]		1.45 [1.29, 1.63]		0.99 [0.87, 1.11]		1.48 [1.15, 1.91]		1.03 [0.87, 1.21]	
Education level: Higher than secondary (ref: None or below primary)	1.96 [1.67, 2.29]		1.69 [1.55, 1.85]		1.06 [0.92, 1.22]		1.37 [1.11, 1.69]		1.07 [0.91, 1.26]	
Employment: In paid employment (ref: Not in paid employment)	1.02 [0.89, 1.16]	0.820	0.95 [0.86, 1.05]	0.334	1.00 [0.93, 1.08]	0.994	0.95 [0.79, 1.14]	0.562	1.07 [0.93, 1.22]	0.350
Marital status: Married or cohabitating (ref: Single)	1.07 [0.93, 1.23]	0.341	0.92 [0.84, 1.01]	0.095	0.94 [0.85, 1.04]	0.225	0.91 [0.75, 1.10]	0.327	0.94 [0.79, 1.12]	0.494
Income: Second tertile (ref: First tertile)	1.05 [0.96, 1.15]	<0.001	1.05 [0.97, 1.14]	<0.001	0.94 [0.86, 1.02]	0.281	1.06 [0.88, 1.28]	0.100	0.76 [0.66, 0.88]	<0.001
Income: Third tertile (ref: First tertile)	1.21 [1.09, 1.34]		1.30 [1.19, 1.41]		0.95 [0.86, 1.04]		1.23 [1.02, 1.48]		0.75 [0.64, 0.88]	
No. of people in the household: 2 (ref: One person)	1.18 [0.83, 1.67]	0.657	0.83 [0.48, 1.41]	0.005	1.27 [1.02, 1.59]	0.020	2.31 [1.39, 3.84]	0.028	1.18 [0.87, 1.60]	0.254
No. of people in the household: 3 (ref: One person)	1.16 [0.82, 1.64]		0.61 [0.36, 1.02]		1.23 [0.99, 1.53]		1.83 [1.12, 2.98]		1.16 [0.85, 1.58]	
No. of people in the household: 4 (ref: One person)	1.12 [0.79, 1.58]		0.67 [0.40, 1.11]		1.11 [0.89, 1.38]		1.86 [1.16, 2.97]		1.00 [0.74, 1.36]	
No. of people in the household: >4 (ref: One person)	1.18 [0.84, 1.67]		0.61 [0.37, 1.00]		1.12 [0.90, 1.39]		1.75 [1.10, 2.78]		1.04 [0.77, 1.41]	

NA—Not Applicable due to unavailable data.

For each South Asia Biobank (SAB) region, the odds ratios were adjusted for all sociodemographic variables shown in the table and additionally for smoking, physical activity, study site, and total energy intake. p-values for age, education, income, and no. of people in the household were computed using likelihood ratio tests, as these variables had more than two categories.

### Sociodemographic correlates of the quantity of ultra-processed food consumption among consumers of ultra-processed food

Similar to the findings on sociodemographic correlates of UPF consumption among all participants, regionally diverse associations were also observed between sociodemographic factors and the quantity of UPF consumption among consumers (n = 37,781) (Table 4). In Bangladesh, UPF consumption was significantly higher among participants aged ≥40 years, while in Sri Lanka, it was significantly higher among younger participants (18–39 years) and no statistically significant associations were observed between age and UPF consumption in India and Pakistan. In all regions except Bangladesh, UPF consumption (among consumers) was higher in women than men. In Bangladesh, Pakistan, and Sri Lanka, rural residents had higher UPF consumption, while in Pakistan, urban residents had higher UPF consumption. In Bangladesh and North India, individuals with higher levels of education had higher UPF consumption, while in Sri Lanka, UPF consumption was significantly lower among those with a higher education level. Being in paid employment was not independently associated with UPF consumption in any region. Across all regions, the quantity of UPF consumed was lower among married or cohabiting individuals compared to single people. In Bangladesh and Pakistan, single-person households consumed more UPFs compared to larger households. Results from the meta-analysis indicated substantial heterogeneity (I^2^ ≥ 75%) in the associations between sociodemographic factors and the quantity of UPF consumption across South Asian regions (Supplementary Figure S3).

**Table 4:** Association between sociodemographic characteristics and the quantity of ultra-processed foods (UPFs) consumed in South Asia, among consumers of UPFs: results from the South Asia Biobank, n = 37,781 (Bangladesh, n = 11,384; Pakistan, n = 6894; Sri Lanka, n = 14,038; North India, n = 2934; South India, n = 2531).

UPFs expressed as %kcal/day	Bangladesh UPF Median 13; IQR 6, 25		Pakistan UPF Median 17; IQR 9, 28		Sri Lanka UPF Median 13; IQR 7, 22		North India UPF Median 15; IQR 8, 27		South India UPF Median 13; IQR 8, 22	
	RD [95% CI]	p value	RD [95% CI]	p value	RD [95% CI]	p value	RD [95% CI]	p value	RD [95% CI]	p value
Age: 40–59 years (ref: 18–39 years)	1.02 [0.99, 1.06]	0.004	0.97 [0.93, 1.01]	0.149	0.96 [0.93, 0.99]	<0.001	0.94 [0.88, 1.00]	0.121	1.02 [0.95, 1.10]	0.409
Age: ≥60 years (ref: 18–39 years)	1.09 [1.03, 1.16]		0.97 [0.92, 1.03]		0.90 [0.86, 0.94]		0.98 [0.90, 1.08]		1.05 [0.96, 1.15]	
Gender: Men (ref: Women)	1.05 [0.99, 1.12]	0.110	0.92 [0.87, 0.97]	0.002	0.96 [0.93, 0.99]	0.011	0.87 [0.80, 0.94]	0.001	0.91 [0.85, 0.97]	0.006
Area of residence: Urban (ref: Rural)	0.96 [0.93, 0.99]	0.028	1.37 [1.29, 1.47]	<0.001	0.90 [0.87, 0.92]	<0.001	NA		NA	
Education level: Primary (ref: None or below primary)	1.06 [1.02, 1.09]	0.093	0.99 [0.94, 1.06]	<0.001	0.96 [0.92, 0.99]	<0.001	1.15 [1.05, 1.26]	<0.001	1.00 [0.93, 1.08]	0.797
Education level: Secondary (ref: None or below primary)	1.06 [0.99, 1.14]		1.05 [0.98, 1.11]		0.92 [0.88, 0.96]		1.24 [1.12, 1.37]		1.04 [0.96, 1.13]	
Education level: Higher than secondary (ref: None or below primary)	1.03 [0.97, 1.09]		1.12 [1.07, 1.17]		0.89 [0.85, 0.94]		1.20 [1.11, 1.31]		1.04 [0.96, 1.12]	
Employment: In paid employment (ref: Not in paid employment)	0.98 [0.93, 1.04]	0.513	0.96 [0.91, 1.01]	0.141	1.02 [0.99, 1.05]	0.276	1.01 [0.94, 1.08]	0.812	1.03 [0.96, 1.09]	0.433
Marital status: Married or cohabitating (ref: Single)	0.94 [0.88, 0.99]	0.044	0.91 [0.86, 0.95]	<0.001	0.95 [0.91, 0.98]	0.005	0.94 [0.88, 1.01]	0.094	0.92 [0.84, 0.99]	0.040
Income: Second tertile (ref: First tertile)	1.04 [0.99, 1.08]	0.383	1.06 [1.01, 1.11]	0.017	0.94 [0.91, 0.97]	<0.001	0.91 [0.85, 0.98]	0.035	0.97 [0.91, 1.04]	0.772
Income: Third tertile (ref: First tertile)	1.02 [0.98, 1.06]		1.06 [1.01, 1.11]		0.96 [0.93, 0.99]		0.97 [0.90, 1.04]		0.98 [0.91, 1.06]	
No. of people in the household: 2 (ref: One person)	0.98 [0.82, 1.16]	0.034	1.09 [0.83, 1.42]	0.029	0.99 [0.91, 1.08]	0.001	0.99 [0.80, 1.22]	0.403	1.11 [0.95, 1.30]	0.326
No. of people in the household: 3 (ref: One person)	0.94 [0.79, 1.12]		0.95 [0.73, 1.24]		1.00 [0.92, 1.09]		1.09 [0.88, 1.35]		1.05 [0.90, 1.23]	
No. of people in the household: 4 (ref: One person)	0.89 [0.75, 1.06]		0.92 [0.71, 1.18]		0.98 [0.90, 1.07]		1.08 [0.88, 1.33]		1.04 [0.87, 1.21]	
No. of people in the household: >4 (ref: one person)	0.93 [0.78, 1.09]		0.92 [0.72, 1.18]		0.94 [0.86, 1.02]		1.05 [0.86, 1.29]		1.02 [0.88,1.19]	

NA—Not Applicable due to unavailable data.

Values presented are relative difference (RD) and 95% CI.

For each South Asia Biobank (SAB) region, the relative differences were adjusted for all sociodemographic variables shown in the table and additionally for smoking, physical activity, study site, and total energy intake. p-values for age, education, income, and no. of people in the household were computed using likelihood ratio tests, as these variables had more than two categories.

## Discussion

In this study of 60,714 participants in South Asia, three in four adults in Bangladesh, North India, and Sri Lanka reported consumption of UPFs in the previous 24-h and in Pakistan and South India, two in five did. Across all five regions, unprocessed and minimally processed foods dominated dietary intake while UPFs contributed to 11% of total energy intake on average. Associations with UPF consumption varied across regions for age, gender, and education. The observed variations highlighted the intricacies of sociodemographic influences on UPF consumption across South Asian countries. Our findings of regional consumption of specific UPFs, such as biscuits, breakfast cereals, sweetened beverages, and salty snacks, provide valuable insights for targeted interventions.

While the NOVA classification has been suggested to be specific, coherent, clear, comprehensive, and workable,^26^ its application to South Asian diets required certain contextual decisions or rules that were standardised for application of NOVA to foods commonly consumed in South Asia. Our classification scheme (Supplementary Table S1) could be a useful starting point to enable application of the NOVA classification in South Asia and other similar contexts. A study conducted in Delhi, India applied the NOVA classification to 24-h dietary recall data from adolescents aged 12–16 years (n = 1030), reporting an average UPF contribution of 16% to total energy intake. However, the study lacked clarity on the method of application of the NOVA classification framework.^27^

Our observation that minimally processed foods make the highest contribution to dietary intake aligns with previous literature from South Asia.^28^ In the current analyses in SAB, across all participants (including consumers and non-consumers of UPFs), UPFs constituted a mean of 83 g per day. This was higher than previously reported by the PURE study, which included data from three of the four South Asian countries in the SAB (Bangladesh, India, and Pakistan), reporting a lower mean UPF intake of 14.5 g per day.^12^ One possible reason for this difference could be the use of different dietary assessment methods used in the two studies. The PURE study used food frequency questionnaires (FFQ), while our study used South Asia specific 24-h dietary recalls. An FFQ with a pre-specified list of food items may underestimate UPF consumption than our detailed 24-h recalls with open-ended reporting that can capture dietary items more comprehensively.^29^ Moreover, dietary data in the PURE study was collected nearly a decade ago and diets have been changing rapidly in developing countries of South Asia. Estimated mean daily energy intake in the SAB were largely comparable to other findings from Sri Lanka^30^ and Pakistan^31^ but lower in India^32^ and Bangladesh,^33^ although this should be placed in the context of differences in study population, sample sizes, and study period. Much of our data was collected during the COVID-19 pandemic during which dietary intakes by the SAB participants may have been influenced due to lockdowns and variable degrees of economic hardship in the region, making it difficult to draw direct comparisons.

Despite the low UPF intakes reported in our study, the recent increase in UPF sales in South Asia is a cause for concern.^14^^,^^15^^,^^34^ Euromonitor market sales data showed that Asian countries had the highest annual growth rate of UPF sales^15^ and there was an increase of over 200% in UPF sales between 2001 and 2016 in some South Asian countries e.g., India and Pakistan.^14^ A recent World Health Organization report on the growth of UPFs in India highlighted sweet biscuits as the leading product in terms of retail sales,^34^ mirroring our finding that biscuits were the most prevalent UPF across all SAB regions, including India.

We identified only two published studies on the sociodemographic factors influencing UPF consumption in South Asia, one each from Bangladesh^17^ and India^18^ but both were limited due to the lack of population representativeness. Specifically, the study from Bangladesh included only rural adolescents (n = 2463), it did not assess associations with total UPF intake but focused on four specific types of UPF consumption, and the sociodemographic variables were limited to gender, household wealth, and maternal and adolescent education.^17^ The study from India included only urban young adults (n = 630) recruited from colleges, and it restricted analysis to high UPF consumption defined as >13% of energy intake from UPFs, without any justification for this definition.^18^ Thus, prior research on the role of sociodemographic factors on UPF consumption in South Asia has been limited.

A systematic review on the relationship between sociodemographic characteristics and UPF consumption, based on nationally representative cohorts, found a consistent association between younger age and higher UPF consumption across 17 countries.^16^ However, in SAB, mixed findings were observed for the association between age and quantity of UPF consumption. In Bangladesh, UPF consumption was higher among older participants, whereas in Sri Lanka, it was higher among younger participants, and no statistically significant associations were observed in India and Pakistan. Notably, the abovementioned systematic review did not include studies from South Asian countries as no relevant published studies were found.^16^ Findings from non-nationally representative studies from other Asian countries have also shown mixed associations with age. For example, in Indonesia, younger age was associated with higher UPF consumption whereas in Japan, UPF consumption did not vary by age.^35^^,^^36^ This heterogeneity reinforces the value of regionally-specific evidence.

One consistent finding across all regions except Bangladesh, was that women were both more likely to consume UPFs and consumed higher quantities of UPFs compared to men. This finding contrasts with studies from other Asian countries, where UPF consumption showed no association with gender or was higher among men. In Japan and Taiwan,^35^^,^^37^ there was no difference in UPF consumption by gender, whereas in China and Singapore, men were more likely to consume higher quantities of UPFs.^38^^,^^39^ One possible explanation for this observed disparity could be gender differences in snacking behaviour in South Asian countries. For instance, a study among college students in Kathmandu reported higher consumption of junk foods, sweets, and bakery products among women compared to men.^40^ Similarly, a study conducted among adults in North and South India found that snack consumption (e.g., bakery products, packaged salty snacks, sweets) was higher among women than men.^41^ These patterns suggest that snacking preferences may contribute to the higher intake of UPFs among women in South Asia. However, more evidence is needed from across the region to better understand gender differences in snacking behaviour, particularly in relation to UPF consumption.

In the current study, income was positively associated with UPF consumption in Bangladesh, Pakistan, and North India. This is consistent with findings from Brazil but contrasts with observations in Canada.^42^ This discrepancy may stem from the observed greater affordability of UPFs in more developed countries,^43^ indicative of a social transition in consumption from higher to lower socioeconomic groups as a country's income increases.^44^ While income was positively associated with UPF consumption, there was no observed association between employment status and UPF consumption. This could be because income data was collected as household income whereas employment was recorded at individual-level. In Bangladesh, Pakistan, and North India, education was positively associated with UPF consumption, consistent with similar observations in middle-income countries like Brazil^45^ and Mexico.^46^ In Pakistan, urban residents reported consuming more UPFs than rural residents, in line with findings from Colombia^47^ and Mexico.^46^ Conversely, rural residents in Bangladesh and Sri Lanka reported higher UPF consumption, a divergent finding that may be potentially explained by market adaptation strategies targeting rural populations. In emerging markets, UPF and beverage manufacturers often use strategies like smaller package sizes and lower prices to make UPFs more affordable and appealing to low-income and rural consumers, as seen in China.^48^ While our study did not include data on food pricing or marketing exposure, such information is crucial for understanding drivers of UPF consumption and designing effective policy responses. Future research should consider incorporating data on food marketing practices and affordability to inform interventions aimed at creating healthier food environments.

Our findings indicate differences in correlates of UPF consumption across sociodemographic characteristics and across regions of South Asia, highlighting the need for caution when generalising findings from one region to another or to South Asia as a whole. This emphasises the need for country or region-specific data to understand the sociodemographic influences on UPF consumption. The current research also indicates distinct associations of sociodemographic factors in the total population and among consumers only (of UPFs). Given that our study only included individuals who self-identified as being of South Asian ethnicity, the findings may not be generalisable to non–South Asian ethnic populations residing in the region.

Our study is the first to investigate a wide range of sociodemographic correlates of UPF consumption in a large sample of South Asian adults. Our use of the detailed 24-h diet recall method with a South Asia-specific foods database allowed a more thorough exploration into the main types of UPFs consumed in five regions of South Asia, in a standardised manner. The large sample size of this study provides adequate precision in our estimates for UPF consumption in the total sample and among consumers only.

Our study has limitations. As this was a cross-sectional observational study, it is not possible to infer causality, but it provides evidence for hypothesis generation. Despite our efforts for a representative recruitment strategy, the recruited samples from India and Pakistan may not be entirely representative of the local population though in Bangladesh and Sri Lanka we were able to recruit a more nationally representative sample. A single 24-h diet recall was used, precluding estimation of habitual dietary consumption without within-person variation, however, it allowed for detailed ascertainment of all food and drinks consumed and the estimations of average population consumption.^29^ Although we used a single 24-h dietary recall, which may be influenced by the day of the week, we conducted a sensitivity analysis adjusting for whether the recall was completed on a weekday or weekend day. The results were unchanged, suggesting minimal impact of day of the week on the association between sociodemographic factors and UPF consumption (data not shown). Similar to other self-report dietary assessment methods, e.g., with a FFQ, the accuracy of responses may be limited by participant memory, misperception about portion sizes, and social desirability bias.^49^ The Hawthorne effect may have modified participant responses due to the presence of an interviewer, with possible underestimation of UPFs.^50^ In the SAB, we collected detailed information on dietary consumption, however, limited details indicative of food processing were available. For example, information on place of meal preparation and or consumption, and brand names were not collected which may lead to misclassification of food items. To reduce this possibility, researchers adhered to a standardised protocol to classify items into NOVA groups, resulting in a strong inter-rater agreement of over 86% in this study. While disaggregating total UPF consumption into specific categories could offer additional insight, the intake of most UPF types such as sugar-sweetened beverages was extremely low across all study sites (median = 0 kcal/day) (Table 2). Consistent with our findings, a study estimating sugar-sweetened beverage intake among adults in South Asia between 1990 and 2018 reported consumption of less than one serving per week.^51^ As a result, meaningful subgroup analyses by UPF subtype and sociodemographic characteristics were not feasible, limiting our ability to explore differential consumption patterns of specific UPF categories. Another limitation of this study is the lack of analysis on the contribution of UPFs to nutrient intakes such as protein, vitamins, minerals, added sugar, and sodium. While this was beyond the current scope that focused on understanding the sociodemographic correlates of UPFs, future work will involve generating and analysing nutrient composition data for UPFs in South Asia to better understand their nutritional impact and health implications.

Our findings of the multifaceted nature of sociodemographic influences on UPF consumption in South Asia emphasise the need for region-specific data for effective public health interventions. General approaches in nutrition policy have included strategies such as clear nutrition labelling, menu labelling, and restricting television advertisements, but further research is needed into specific approaches targeted at reducing UPF consumption in South Asia. It is possible that a specific, targeted intervention may work for a particular type of UPF consumption. For example, potentially, public health messages could specifically encourage individuals to limit biscuit consumption in South Asia, if further research confirmed our findings and was extended to also investigate the association between biscuit consumption (as part of UPF consumption) and health outcomes. Further studies are needed to confirm our overall findings and to understand the barriers to consumption of less processed foods. Additionally, conducting similar studies in younger population groups, including, infants, children, and adolescents, will provide valuable public health insights.

To our knowledge, this is the first study to conduct a comprehensive examination of the sociodemographic correlates of UPF consumption in a large sample of South Asian adults, using detailed dietary consumption data in four South Asian countries. Further research in South Asia will help to confirm and expand our findings on the drivers of UPF consumption. Combined with studies on the link between UPF consumption and health outcomes, this research will be vital in shaping effective public health strategies in South Asia as it navigates through economic growth, urbanisation, globalisation, and dietary transition.

## Contributors

JCC, RMA, KIK, VJ, AK, PK, MKM, and NGF are investigators and collaborators of the South Asia Biobank and conceptualised the study and enabled data collection. AL classified foods based on the NOVA classification system as a second coder with DB. DB analysed the data and drafted the manuscript. NGF, JA, and FI supervised the work and critically reviewed previous drafts. All authors read, commented on, and approved the final manuscript.

## Data sharing statement

The South Asia Biobank data are available to researchers upon request. Data requests should be made via email to the study Steering Committee (john.chambers@imperial.ac.uk).

## Editor note

The Lancet Group takes a neutral position with respect to territorial claims in published maps and institutional affiliations.

## Declaration of interests

JCC is supported by the Singapore Ministry of Health's National Medical Research Council under its Singapore Translational Research Investigator (STaR) Award (NMRC/STaR/0028/2017). NGF, JA, DB, and FI acknowledge core support from the Medical Research Council Epidemiology Unit (grant no. MC UU 00006/3 and MC_UU_00006/7). AL acknowledges funding by the Gates Cambridge Trust. DB and NGF acknowledge funding from the NIHR Cambridge Biomedical Research Centre theme on nutrition, obesity, endocrinology and metabolism (NIHR203312) and NGF is an NIHR Senior Investigator (NIHR202397). JA acknowledges receiving institutional research grants from the Medical Research Council for the present manuscript, and within the past 36 months, from UKRI, NIHR Policy Research Programme, NIHR Public Health Research Programme, NIHR School of Public Health Research, Global Alliance for Chronic Disease, ESRC, BBSRC, and Canadian Institutes of Health Research. JA also acknowledges receiving consulting fees from the Food Foundation for overseeing commissioned research, and payment for education from Bloomberg Philanthropies via JBS Executive Education, both paid to her institutional account. Additionally, within the past 36 months, she received reimbursement for travel, conference fees, and accommodation from the European Association for the Study of Obesity for a panel speaker engagement. Her unpaid advisory roles include Chair of the NIHR Healthy Weight Policy Research Unit advisory board, Member of the NIHR Policy Research Programme project (Healthy Start) advisory board, Member of the Public Health Scotland (HFSS evaluation) advisory group, and Member of the Best Food Forward (school food) advisory group. JA holds a leadership role on the Scientific Advisory Committee on Nutrition, receiving institutional payments for meeting preparation.

The views expressed are those of the authors and not necessarily those of the NIHR or the Department of Health and Social Care. We declare no other competing interests.
