# Supplementary material for: Quantification of regional variation in ultra-processed food consumption and its sociodemographic correlates across Bangladesh, India, Pakistan, and Sri Lanka: insights from the South Asia Biobank
Source: Lancet Reg Health Southeast Asia. 2025 Jul 18;39:100633. doi: 10.1016/j.lansea.2025.100633 (PMC12302672; doi:10.1016/j.lansea.2025.100633)
Supplement: Supplementary Tables and Figures [file mmc1.pdf]

Quantification of regional variation in ultra-processed food consumption and its sociodemographic correlates across Bangladesh, India, Pakistan, and Sri Lanka: insights from the South Asia Biobank

D Bhagtani et al.

**Supplementary materials**	**Page**
Supplementary Table S1: The NOVA classification framework	2
Supplementary Figure S1: Histogram of ultra-processed food (UPFs)	3
Supplementary Figure S2: Odds ratio for the association between sociodemographic characteristics and ultra-processed foods (UPFs) in South Asia, among all participants including consumers and non-consumers of UPFs	4
Supplementary Figure S3: Association between sociodemographic characteristics and the quantity of ultra-processed foods (UPFs) consumed in South Asia, among consumers of UPFs	5
References	6

Supplementary Table S1: The NOVA classification framework

**Group 1. Unprocessed and minimally processed foods**	
Definition	Unprocessed foods are edible parts of animals (muscle, offal, eggs, milk), plants (seeds, fruits, leaves, stems, roots), fungi, algae, and water. Minimally processed foods are natural foods altered by processes such as removal of inedible or unwanted parts, drying, crushing, grinding, fractioning, filtering, roasting, boiling, pasteurisation, refrigeration, chilling, freezing, placing in containers and vacuum packaging.
Purpose	To preserve natural foods, make suitable for storage or extend shelf life, and make foods safer, edible or more pleasant to consume.
Characteristics	No substances are added to the original food (such as salt, sugar or fats). Vitamins and minerals may be added with the purpose of replacing nutrients lost during processing. May infrequently contain additives to preserve the properties of the original food (such as antioxidants and stabilisers). Minimally processed foods also include culinary preparations such as freshly prepared vegetable, meat or fish-based curries and rice dishes, chapatti, soups, and chutneys made using unprocessed and minimally processed foods containing small quantities of oils, fats, salt, sugar, etc.
Examples	Fresh fruits, legumes, vegetables, roots, and tubers; fresh or pasteurised fruit or vegetable juices with no added sugar, sweeteners, or flavours; squeezed, chilled, frozen and dried fruits; vacuum-packed vegetables with added antioxidants; nuts and seeds with no added salt, sugar or oil; mix of fruits, nuts, cereals (granola) with no added salt, sugar, honey or oil; grains such as rice, beans, lentils, corn and wheat; grits, flakes or flours made from corn, wheat, oats or cassava (fortified or not with iron or folic acid); pasta and couscous made with flours, flakes or grits and water with no salt or oil added; fresh, chilled or frozen meat, poultry, fish, seafood (whole, in the form of steaks, fillets or other cuts); fresh or pasteurised eggs; fresh cottage cheese or paneer; fresh, pasteurised, powdered or ultra-pasteurised milk with added stabilisers; plain yogurt with no added sugar or artificial sweeteners; fresh or dried spices and herbs; tea, coffee, and drinking water.
**Group 2. Processed culinary ingredients**	
Definition	Substances obtained directly from unprocessed and minimally processed foods or from nature by processes such as pressing, refining, grinding, milling and drying.
Purpose	To prepare, cook, and season unprocessed and minimally processed foods.
Characteristics	They are not meant to be consumed by themselves but are typically used in combination with Group 1 foods to prepare homemade dishes and meals. May contain additives used to preserve the original properties of foods. Added vitamins or minerals may be present.
Examples	Mined or seawater salt, iodised or not; cooking salt with added anti-humectants; sugar, jaggery, treacle, and molasses obtained from cane or beet, honey extracted from combs, syrup from maple trees; vegetable oils crushed from olives or seeds, with or without added antioxidants; butter, ghee, and lard obtained from milk or pork, salted or not; starches extracted from corn and other plants; vinegar made by acetic fermentation of wine or other alcoholic drinks, with or without added preservatives.
**Group 3. Processed foods**	
Definition	Foods made by adding Group 2 ingredients (such as sugar, oil, salt) to Group 1 foods. Processes include various preservation or cooking methods, and non-alcoholic fermentation (in the case of breads and cheeses).
Purpose	To increase durability and enhance sensory qualities of Group 1 foods.
Characteristics	May contain additives to preserve the original properties of foods or to resist microbial contamination.
Examples	Artisanal breads and cheeses; canned and bottled vegetables, fruits, legumes and fish, with added antioxidants or not; fruits in syrup, with added antioxidants or not; salted or sugared nuts, seeds and dried fruits; salted, cured, dried or smoked meats with or without added preservatives.
**Group 4. Ultra-processed foods**	
Definition	Industrial formulations made mostly or entirely from substances derived from foods and additives. Contain small if any proportion of whole foods. Processes include several industrial methods with no domestic equivalents such as extrusion, moulding, hydrogenation, hydrolysation and pre-processing for frying.
Purpose	To create convenient and ready-to-consume food products, with long shelf life, liable to displace unprocessed or minimally processed foods as well as freshly prepared dishes.
Characteristics	Typically contains five or more ingredients, and substances not commonly used in culinary preparations, such as hydrolysed protein, modified starches, hydrogenated or interesterified oils, etc. They are hyper-palatable, sold in attractive packages, intensively marketed, highly profitable food products. Contain additives used to imitate sensorial qualities of unprocessed or minimally processed foods, or to disguise undesirable qualities of the final product such as colourants, flavourings, artificial sweeteners, emulsifiers, humectants, sequestrants, and firming, bulking, de-foaming, anti-caking and glazing agents. It includes products made solely of Group 1 or Group 3 foods with added cosmetic or sensory intensifying additives.
Examples	Breads with added emulsifiers and mass-produced packaged breads and buns; breakfast cereals and puffed rice, energy and cereal bars; margarines and spreads; reconstituted meat products, such as poultry and fish sticks, sausages, burgers, hot dogs and bacon; meat and chicken extracts and instant sauces; powdered and packaged instant soups and noodles; ready to heat pre-prepared pies, pasta, pizza, dishes, and desserts; sweet or savoury packaged snacks, cookies, pastries, cakes, cake mixes, chocolate, candies, ice-cream, etc.; fritters and other deep-fried savoury or sweet items; carbonated drinks, energy drinks, fruit drinks, milk drinks, and cocoa drinks; fruit yogurts and plain yogurt with added artificial sweeteners; health and slimming products such as powdered or fortified meal and dish substitutes; infant formulas, follow-on milks, and other baby products.

Source: adapted from Nardocci et al., 2019,[1] Moubarac et al., 2017,[2] and Monteiro et al., 2018.[3]

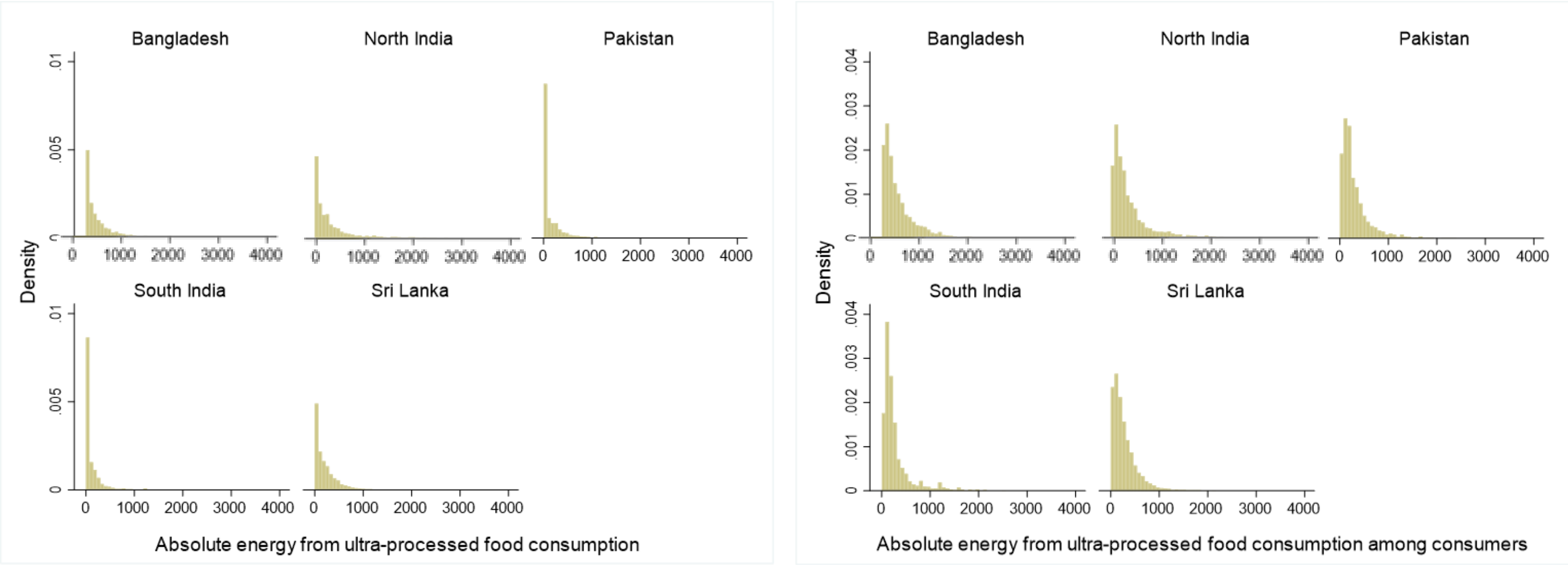

Supplementary Figure S1: Histogram of ultra-processed food (UPFs) consumption across all South Asia Biobank regions, in all participants (consumers and non-consumers of UPFs) (Left panel) and separately among consumers of UPFs (right panel), expressed in terms of absolute energy intake from UPFs.

	Bangladesh	Pakistan	Sri Lanka	North India	South India	Pooled	I^2
**Proportion of consumers**	74·8%	41·0%	75·3%	74·3%	41·4%	62·2%	-
**Age (in years)**	1·01***	0·99**	0·99***	1·01*	1·00	1.00	92·9%***
**Gender: Men (ref: Women)**	1·35***	0·90*	0·72***	0·85	0·82**	0·83	90·1%***
**Area of residence: Urban (ref: Rural)**	1·06	1·05	0·88***	NA	NA	0·96	85·4%***
**Education level: Primary (ref: None or below primary)**	1·43***	1·28***	0·99	1·24	1·06	1·18	86·6%***
**Education level: Secondary (ref: None or below primary)**	1·67***	1·45***	0·97	1·53**	1·04	1·17	89·8%***
**Education level: Higher than secondary (ref: None or below primary)**	2·01***	1·69***	1·04	1·40***	1·10	1·35	93·3%***
**Employment: In paid employment (ref: Not in paid employment)**	1·02	0·94	1·00	0·94	1·06	0·99	0·0%
**Marital status: Married or cohabitating (ref: Single)**	1·10	0·89*	0·97	0·93	0·94	0·95	33·5%
**Income: Second tertile (ref: First tertile)**	1·06	1·04	0·94	1·07	0·76***	0·97	81·5%***
**Income: Third tertile (ref: First tertile)**	1·22***	1·30***	0·95	1·23*	0·75***	1·05	93·3%***
**No. of people in the household**	1·01	0·99**	0·98*	0·99	0·98	0·99	13·8%

Positive association (odds ratio >1)

Negative association (odds ratio <1)

Supplementary Figure S2: Odds ratio for the association between sociodemographic characteristics and ultra-processed foods (UPFs) in South Asia, among all participants including consumers and non-consumers of UPFs: results from the South Asia Biobank, n=60,714 (Bangladesh, n=15,212; Pakistan, n=16,804; Sri Lanka, n=18,634; North India, n=3,948; South India, n=6,116). Red colour indicates positive associations and blue indicates negative associations. NA: Not Applicable due to unavailable data. * p<0·05, ** p<0·01, *** p<0·001. For each SAB region, the odds ratios were adjusted for all sociodemographic variables shown in the figure and additionally for smoking, physical activity, study site, and total energy intake. For the meta-analysis, estimates from each of the SAB regions were pooled using a fixed effects model, weighted by the inverse of regional specific variances. The I^2 statistic indicates substantial heterogeneity between the regions (>=75%).

	Bangladesh	Pakistan	Sri Lanka	North India	South India	Pooled	I^2
Median UPF consumption (IQR)	13 (6,25)	17 (9,28)	13 (7,22)	15 (8,27)	13 (8,22)	14 (7,24)	-
Age (in years)	1·00***	0·99	0·99***	0·99	1·00	1·00	91·1%***
Gender: Men (ref: Women)	1·05	0·93**	0·96*	0·88**	0·91**	0·95	75·7%***
Area of residence: Urban (ref: Rural)	0·96*	1·36***	0·90***	NA	NA	0·94	98·0%***
Education level: Primary (ref: None or below primary)	1·06**	0·99	0·95*	1·15**	1·00	1·01	81·1%***
Education level: Secondary (ref: None or below primary)	1·07*	1·04	0·91***	1·25***	1·04	0·99	90·0%***
Education level: Higher than secondary (ref: None or below primary)	1·04	1·12***	0·89***	1·21***	1·04	1·02	93·2%***
Employment: In paid employment (ref: Not in paid employment)	0·98	0·96	1·02	0·99	1·03	1·00	32·3%
Marital status: Married or cohabitating (ref: Single)	0·92**	0·90***	0·96*	0·93*	0·92*	0·93	13·6%
Income: Second tertile (ref: First tertile)	1·03	1·06*	0·94***	0·92*	0·97	0·98	84·6%***
Income: Third tertile (ref: First tertile)	1·01	1·06*	0·96*	0·97	0·97	0·99	68·0%**
No. of people in the household	0·99	0·99*	0·99***	1·00	0·99	0·99	27·2%

Positive association (relative difference >1)

Negative association (relative difference <1)

Supplementary Figure S3: Association between sociodemographic characteristics and the quantity of ultra-processed foods (UPFs) consumed in South Asia, among consumers of UPFs: results from the South Asia Biobank, n=37,781 (Bangladesh, n=11,384; Pakistan, n=6,894; Sri Lanka, n=14,038; North India, n=2,934; South India, n=2,531). Values presented are relative difference (RD) and 95% CI. Red colour indicates positive associations and blue indicates negative associations. NA: Not Applicable due to unavailable data. * p<0·05, ** p<0·01, *** p<0·001. For each SAB region, the relative differences were adjusted for all sociodemographic variables shown in the figure and additionally for smoking, physical activity, study site, and total energy intake. For the meta-analysis, estimates from each of the SAB regions were pooled using a fixed effects model, weighted by the inverse of regional specific variances. The I^2 statistic indicates substantial heterogeneity between the regions (>=75%).

References:

1. Nardocci M, Leclerc BS, Louzada ML, Monteiro CA, Batal M, Moubarac JC. Consumption of ultra-processed foods and obesity in Canada. Can J Public Health. 2019 Feb;110(1):4–14.

2. Moubarac JC, Batal M, Louzada ML, Martinez Steele E, Monteiro CA. Consumption of ultra-processed foods predicts diet quality in Canada. Appetite. 2017 Jan;108:512–20.

3. Monteiro CA, Cannon G, Moubarac JC, Levy RB, Louzada MLC, Jaime PC. The UN Decade of Nutrition, the NOVA food classification and the trouble with ultra-processing. Public Health Nutr. 2018 Jan;21(1):5–17.
